# Special Issue “Tryptophan in Nutrition and Health 3.0”

**DOI:** 10.3390/ijms26178443

**Published:** 2025-08-30

**Authors:** Burkhard Poeggeler, Sandeep Kumar Singh, Kumar Sambamurti, Miguel A. Pappolla

**Affiliations:** 1 Department of Physiology, Johann-Friedrich-Blumenbach Institute for Zoology and Anthropology, Faculty of Biology and Psychology, Georg August University Göttingen, Zappenburg 2, D-38524 Sassenburg, Germany; 2 Department of Medical Biotechnology, All India Institute of Medical Sciences (AIIMS) Nagpur, Nagpur 441108, Maharashtra, India; sandeeps.bhu@gmail.com; 3 Department of Neurobiology, Medical University of South Carolina, 173 Ashley Avenue, BSB 403, Charleston, SC 29425, USA; sambak@musc.edu; 4 Mitchell Center for Neurodegenerative Diseases, John Sealy School of Medicine, Department of Neurology, University of Texas Medical Branch, 301 University Boulevard, Galveston, TX 77555, USA; mapappol@utmb.edu

Tryptophan is the rate-limiting essential amino acid and thus a building block of life that is needed for protein synthesis. Tryptophan is the precursor to serotonin, melatonin, protective indole acids, reactive indole compounds, kynurenines, and niacin, as shown in Figure 1.

Current research on tryptophan metabolism has revealed the central role of tryptophan and its metabolites as molecular modulators of physiology and development. The Special Issue “Tryptophan in Nutrition and Health 3.0” examined the key tryptophan pathways and their molecular targets. The discovery of a broad range of bioactive compounds derived from tryptophan can enable a better understanding of the unique role of this amino acid in disease prevention and treatment. This Special Issue enriches our understanding of tryptophan’s multifaceted roles—from metabolic and microbial pathways to animal husbandry and human health. Taken together, the seven contributions (five research articles and two review articles) lay substantive groundwork for future research targeting nutritional modulation and biotechnology-based production of bioactive compounds, as well as translational applications in animal and human systems.

The microbial biosynthesis of tryptophan metabolites, such as 5-hydroxytryptophan and related indoleamines using the yeast strain STG S101, enables the production of significant amounts of these agents that are used in supplements for managing various mood-related disorders, including depression and insomnia [1]. Their production via chemical synthesis suffers from serious drawbacks, such as the generation of dangerous contaminants, toxic byproducts, and low yields. Optimizing medium composition enabled sufficient production of 5-HTP and serotonin. No melatonin was detected. This approach allows for a promising and sustainable biotechnological route for 5-hydroxytryptophan and indolamine production. The utilization of Saccharomyces cerevisiae presents a promising avenue, offering scalability, sustainability, reduced environmental impact, and feasibility for large-scale production. The yeast-based microbial production introduces an eco-friendly platform for producing neuroactive compounds, potentially mitigating reliance on chemical synthesis or plant extraction.

The supplementation of rumen-protected L-tryptophan in early-lactating Holstein cows under heat stress increased dry matter intake, milk yield, and content. Decreased physiological stress markers such as heart rate and rectal temperature, and enhanced expression of heat-shock proteins, indicated improved adaptation and enhanced resilience to the thermal challenge [2]. The enhanced productivity with improved physiological indicators, blood characteristics, and gene expression in the peripheral blood mononuclear cells of early lactating Holstein cows under heat-stress conditions suggests that supplementation objectively relieved stress in these animals. L-tryptophan supplementation has potential as an animal application and viable solution for combating heat-stress-induced effects on cattle in dairy farming. Rumen-protected L-tryptophan shows tangible benefits for dairy production and animal welfare through alleviating heat-induced discomfort.

Postmenopausal women often suffer from constipation, but the mechanisms that induce constipation and associated symptoms are not fully known. The relationship between some metabolites of tryptophan and the occurrence and severity of abdominal symptoms in postmenopausal women with functional constipation was assessed and compared to that in age-adjusted postmenopausal women without functional constipation [3]. Urinary levels of tryptophan and its metabolites, 5-hydroxyindoleacetic acid, kynurenine, and 3-indoxyl sulfate (indican), were determined. Dysbiosis was assessed by a hydrogen–methane breath test. Women with functional constipation consumed less tryptophan and had a lower urinary level of 5-hydroxyindoleacetic acid but higher levels of kynurenine and 3-indoxyl sulfate compared with controls. The severity of symptoms showed a negative correlation with the 5-HIAA level but a positive correlation with the 3-IS level. Changes in tryptophan metabolism may contribute to functional constipation in postmenopausal women, and dysbiosis may underlie this contribution. All menopausal women had increased levels of exhaled hydrogen and methane. The modulation of tryptophan metabolism has the potential to improve gut motility and associated symptoms. The findings confirm the emerging relevance of tryptophan in gastrointestinal regulation, offering potential dietary approaches to prevention and treatment of constipation and associated symptoms.

L-tryptophan-depleted cells display a marked enhancement in tryptophan uptake facilitated by extracellular tryptophanyl-tRNA synthetase (TrpRS). Tryptophan uptake into TrpRS-overexpressing cells is markedly elevated upon tryptophan starvation [4]. A tryptophan-deficient condition is critical for tryptophan uptake, not only into cells to which TrpRS protein has been added but also into TrpRS-overexpressing cells. Tryptophan-starved human cells overexpressing TrpRS enhance high-affinity tryptophan uptake via enzymatic production of tryptophanyl-AMP. Overexpression of TrpRS mutants, which cannot synthesize tryptophanyl-AMP, prevents the increase in tryptophan uptake, and inhibition of tryptophanyl-AMP synthesis suppresses this uptake. Tryptophanyl-AMP production by TrpRS is critical for high-affinity tryptophan uptake.

Research on the modulatory role of tryptophan in European seabass (Dicentrarchus labrax) on stressful conditions and acute inflammation revealed the role of dietary tryptophan supplementation on fish immune responses during stressful rearing conditions, such as a 15-day exposure to high stocking density and to acute inflammation after intraperitoneal injection of a bacterial pathogen [5]. Tryptophan supplementation supported immune tolerance, ameliorated stress-induced immunosuppression, reduced cortisol levels, and elevated kynurenine pathway gene expression—highlighting the role of tryptophan in managing stress, infection, and immune responses in aquaculture. Supplementation improved the inflammatory response against a bacterial pathogen during stressful conditions, supported by a reduction in plasma cortisol levels, an up-regulation of several immune-related genes at 48 h, and an inversion of the previously observed, stress-induced T-cell suppression. Finally, the involvement of tryptophan catabolism in macrophages was confirmed by the upregulation of genes involved in the kynurenine pathway. This work allows for the development of novel prophylactic protocols during stress, infection, and vaccination. Tryptophan-enriched diets may safeguard fish health in intensive farming environments, enhancing immune resilience.

Aging and the demographic transition pose great challenges to science and society since the prevalence of neurodegenerative diseases affecting the elderly increases with the longer life span. The gastrointestinal microbiota affect cognitive function, neuronal survival, and gut dysbiosis in Parkinson’s disease [6]. Tryptophan is degraded by the microbiota and their human hosts to numerous bioactive compounds with immune- and neuromodulating properties by aryl hydrocarbon receptor stimulation. The microbiota, tryptophan, and the aryl hydrocarbon receptors form a target triad in Parkinson’s disease. A disturbed gut–brain axis allows the bidirectional spread of pro-inflammatory molecules and α-synuclein, which contribute to the development and progression of the disease, whereas a well-balanced gut–brain connection contributes to the generation of anti-inflammatory compounds that enable adaptation and neuroprotection. Tryptophan and its indole and kynurenine metabolites modulate AhR signaling and are prime targets for disease prevention and treatment. Tryptophan and tryptophan pathway modulating pre-, post-, and probiotics are of potential benefit and deserve further experimental and clinical research as disease-modifying tools. The robust crosstalk between the microbiota in the gut and the brain can induce neurotrophic and neuroprotective signaling dependent on stress resilience and immune adaptation.

A review on the source, fate, and analysis of dietary kynurenine pathway metabolites demonstrates that ~96% of dietary tryptophan undergoes conversion via the kynurenine pathway [7]. This work focuses on the occurrence of kynurenines in foods, analytical detection methods, and implications for human health in relation to immune signaling, inflammation, and carcinogenesis. Some kynurenine pathway metabolites are potent agonists of the aryl hydrocarbon receptor involved in metabolic regulation, immune responses, and xenobiotic signaling. The food-based identification of kynurenines and analytical approaches advances our capacity to track the dietary impact on systemic health that depends on regulatory immune metabolites with potent anti-inflammatory and antioxidant effects such as kynurenic acid. Reactive pro-oxidant and pro-inflammatory compounds such as quinolinic acid have also been identified, and their formation must be controlled. Understanding dietary intake and metabolism of kynurenines enables the development of nutritional strategies to modulate inflammation and neurodegenerative risk that can be used to improve health.

## Figures and Tables

**Figure 1:**
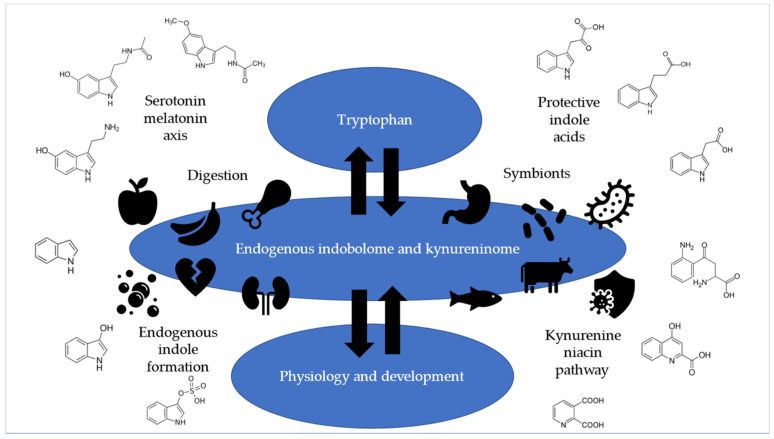
The serotonin–melatonin axis, protective indole acids, endogenous indole formation, and the kynurenine–niacin pathway are dependent on a sufficient supply of tryptophan.
